# Rhegmatogenous retinal detachment in Coats’ disease: a case report

**DOI:** 10.1186/s13256-021-03221-6

**Published:** 2021-12-31

**Authors:** Simanta Khadka, Raghunandan Byanju, Sabina Parajuli

**Affiliations:** Department of Vitreo-Retina, Bharatpur Eye Hospital, Bharatpur, Chitwan Nepal

**Keywords:** Coats’ disease, Rhegmatogenous retinal detachment, Silicon oil, Vitrectomy

## Abstract

**Background:**

Coats’ disease was originally defined as a unilateral idiopathic exudative retinopathy in young males, characterized by abnormal retinal vascular telangiectasia with intraretinal and subretinal lipid exudation. The retinal detachment is usually exudative. Herein, we describe a case of rhegmatogenous retinal detachment with detectable retinal break in a patient with Coats’ disease.

**Case presentation:**

A 15-year-old Indo-Aryan male patient presented with sudden painless diminution of vision in his right eye of 4 days duration. Upon examination, the anterior segment in both eyes and left fundus was within normal limits. Dilated fundus evaluation of the right eye revealed telangiectasia of the retinal vessels, with subretinal exudation in superotemporal and superonasal quadrants and presence of subretinal fluid in the superotemporal area extending into fovea. There was also presence of single flap horseshoe tear in the superotemporal quadrant at around the 10 o’clock position in the equatorial region, with no secondary changes. The retina was reattached with encircling band buckle combined with vitrectomy and silicon oil tamponade. Seven months post vitrectomy, lenticular opacification developed, for which he underwent silicon oil removal, along with lens aspiration and implantation of foldable intraocular lens. Over the period of 1 year, his best corrected visual acuity improved from 6/60 to 6/18 in the affected eye at the last follow-up visit. The recovery was uneventful following the subsequent surgery.

**Conclusion:**

Coats’ disease has a remarkable diversity in clinical presentation and morphology. The disease can also present with an underlying break, which may not be attributed to any iatrogenic modality. The treatment modalities in coats’ disease should be tailored individually due to the low incidence of the disease and the great variation in severity upon presentation. Prompt management restores the best possible anatomical outcome and maintains good vision.

## Introduction

The first description of Coat disease in 1908 is credited to Scottish Ophthalmologist George Coats [1]. It is defined as an idiopathic exudative retinopathy, characterized by abnormal retinal vascular telangiectasia with intraretinal and subretinal lipid exudation [2]. The condition is sporadic, with no associated systemic abnormalities. The typical presentation is unilateral involvement in young males, with most cases being diagnosed in the first and second decade of life. The severity depends upon the age of the patient, and tends to become more severe with poor visual outcomes, especially in young patients [3].

Coats’ disease has a remarkable diversity in clinical presentation and morphology [2]. The disease is primarily juvenile but can affect adults [4]. It is a progressive condition, which can be asymptomatic in early stages and only incidental during routine ophthalmologic evaluation. The presentation can be varied with a range of signs, the most common being decreased visual acuity followed by strabismus and leukocoria or xanthocoria. Similarly, pain, iris heterochromia, and nystagmus have also been reported as a presenting signs [5].

There is a high incidence of reported retinal detachment in Coats’ disease [6]. The detachment varies from focal retinal elevation to total retinal separation. However, the associated retinal detachment is usually nonrhegmatogenous [7]. Herein, we describe a case of rhegmatogenous retinal detachment with a detectable retinal break, retinal vascular telangiectasia, and subretinal lipid exudation.

## Case presentation

**Fig. 1:**
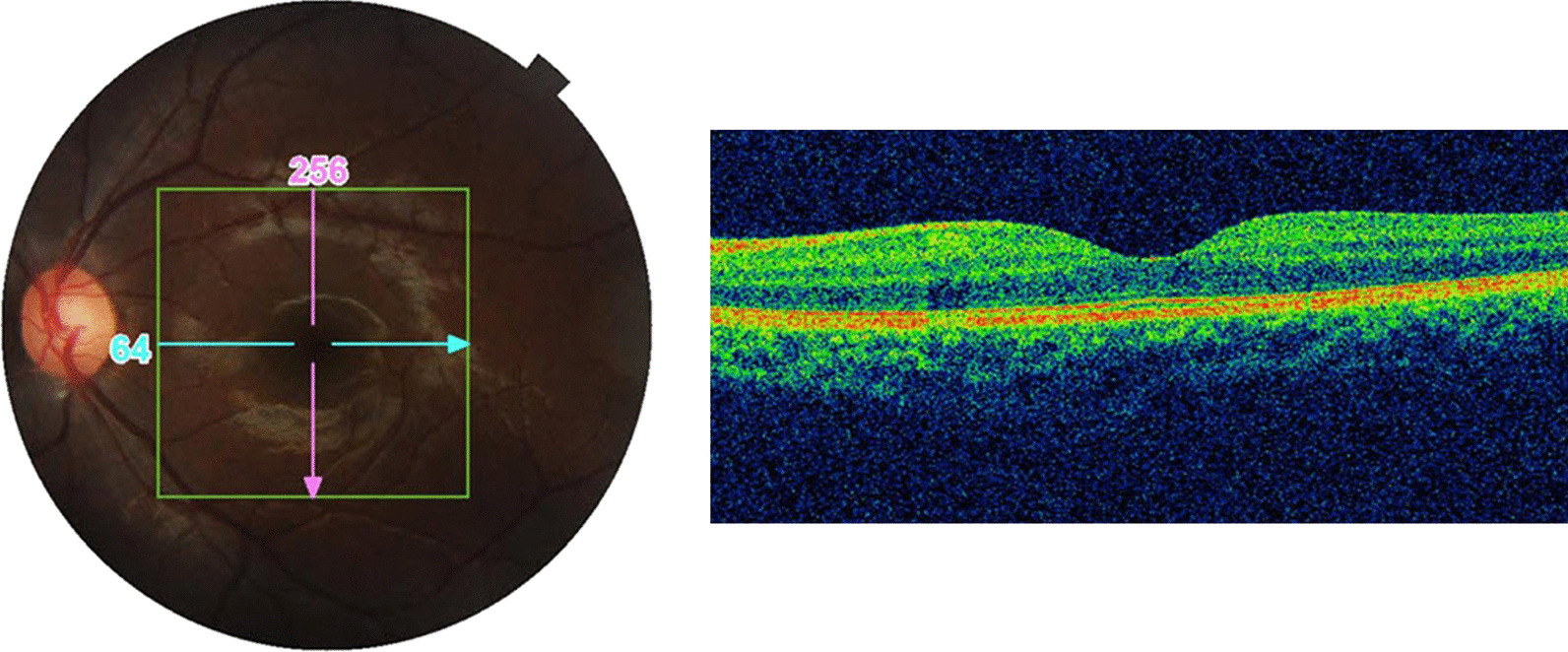
Normal appearance of left eye and optical coherence tomography image of the same eye

**Fig. 2:**
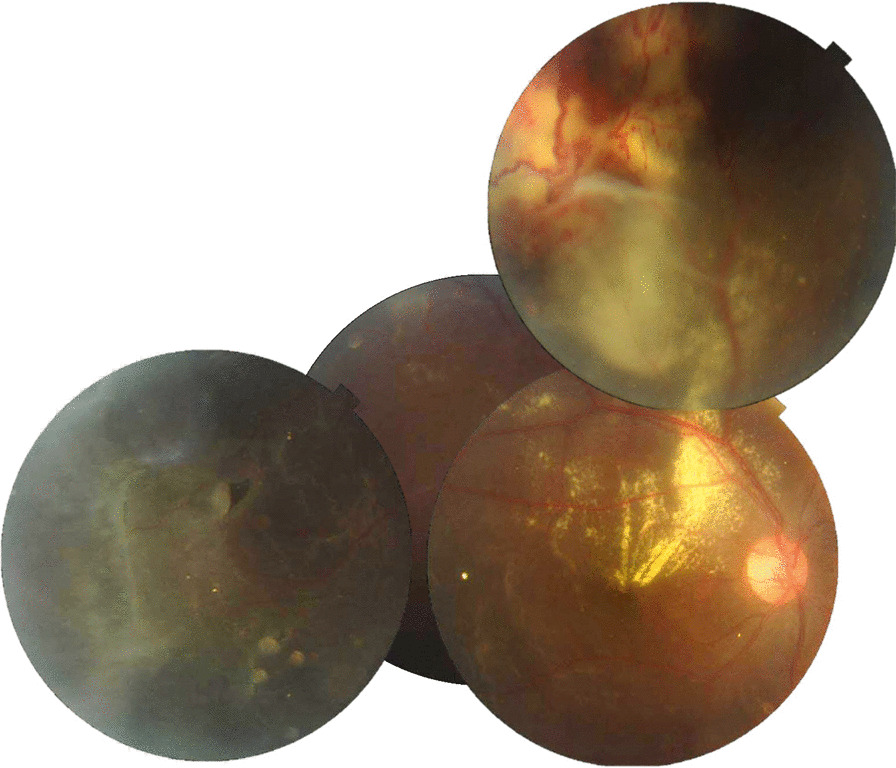
Composite montage of right eye. Exudate is present in the macula. Telangiectasia with lipid exudation in the superior quadrant and a horse shoe tear is visible in the temporal quadrant

A 15-year-old Indo-Aryan male patient presented with a sudden painless diminution of vision in his right eye (RE) of 4 days duration (February 2020). He had no significant ocular history or similar family history. Review of the systems was negative. Ocular examination at presentation revealed a best corrected visual acuity (BCVA) of 6/60 in his RE and unaided visual acuity of 6/6 in his left eye (LE). The intraocular pressure (IOP) were within the normal range, at 15 and 16 mmHg in RE and LE, respectively. Pupillary reactions revealed afferent pupillary defect in RE. Upon slit lamp examination, the anterior segment findings were within the normal limit in both eyes, with a normal fundus in LE (Fig. 1). Dilated fundus evaluation in RE showed telangiectasia of the retinal vessels with subretinal exudation in superotemporal and superonasal quadrants, and presence of subretinal fluid in the superotemporal area extending into fovea. There was also presence of a single flap horseshoe tear in the superotemporal quadrant at around the 10 o’ clock position in the equatorial region, with no secondary changes (Fig. 2).

**Fig. 3:**
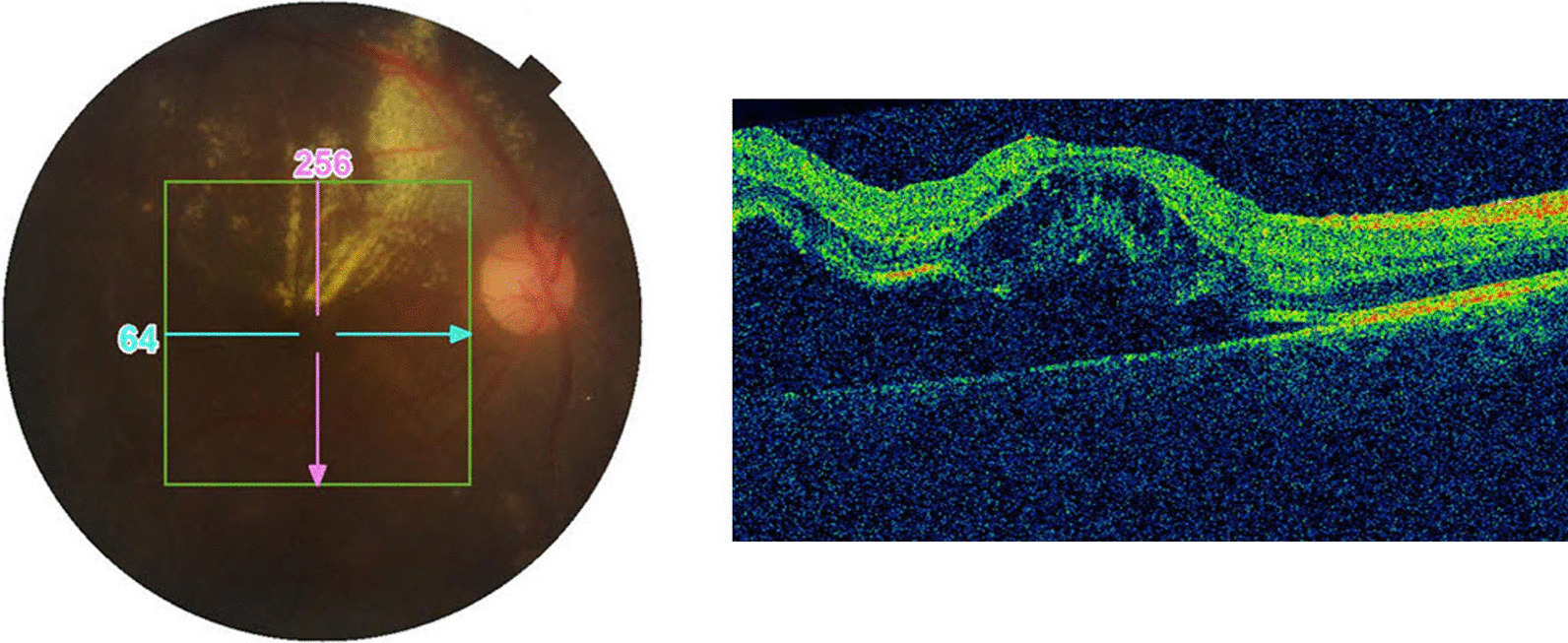
Spectral domain optical coherence tomography of right eye showing lipid exudation with presence of subretinal fluid and retinal detachment

His complete blood counts, viral serology, plain chest roentgenogram, and Mantoux test affirmed normal reports. Spectral Domain Optical Coherence Tomography (SD-OCT) done subsequently revealed hyporeflective signal between the retinal pigment epithelial layer and the neurosensory layer, suggestive of subretinal fluid (Fig. 3). The patient was diagnosed with Coats’ disease with rhegmatogenous retinal detachment after careful exclusion of all other possible differential diagnosis including retinoblastoma, retinopathy of prematurity, familial exudative vitreoretinopathy, and toxocariasis. Surgical management was performed under general anesthesia, with placement of encircling band buckle followed by lens sparing pars plana vitrectomy, endolaser photocoagulation around the telangiectatic vessels, and internal drainage of subretinal fluid. Three hundred sixty degree cryotherapy was applied, and the surgery was concluded with instillation of 1000-centistokes silicone oil. Postoperatively, the patient was treated with oral tablet ciprofloxacin 500 mg twice daily (BD) for 7 days, tablet acetazolamide 250 mg BD for 3 days. Prednisolone acetate drops (gtt) 1% in a tapering fashion, gtt atropine 1%, and gtt moxifloxacin 0.5% was prescribed the day after surgery and continued for 4 weeks.

**Fig. 4:**
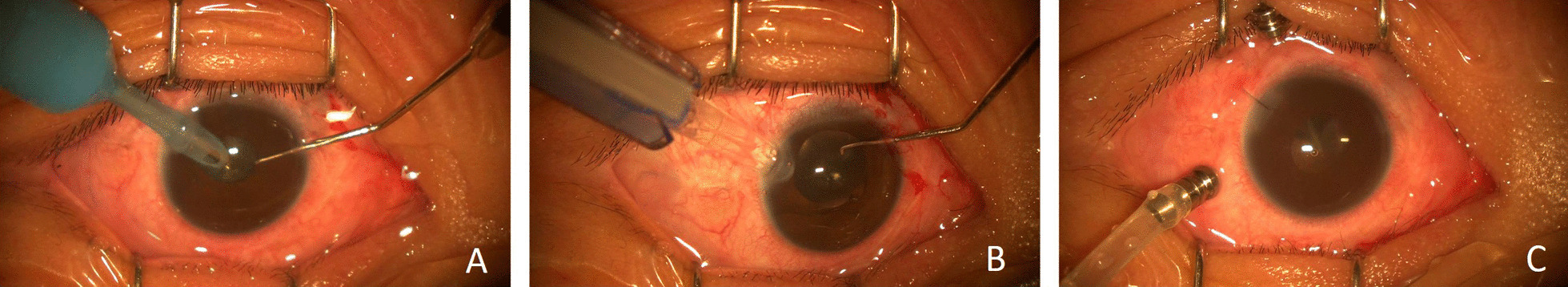
Subsequent surgery following retinal reattachment. **A** Lens aspiration is being performed with co-axial irrigation aspiration probe. **B** Single piece foldable hydrophobic posterior chamber intraocular lens is being injected into the capsular bag. **C** Removal of silicon oil

**Fig. 5:**
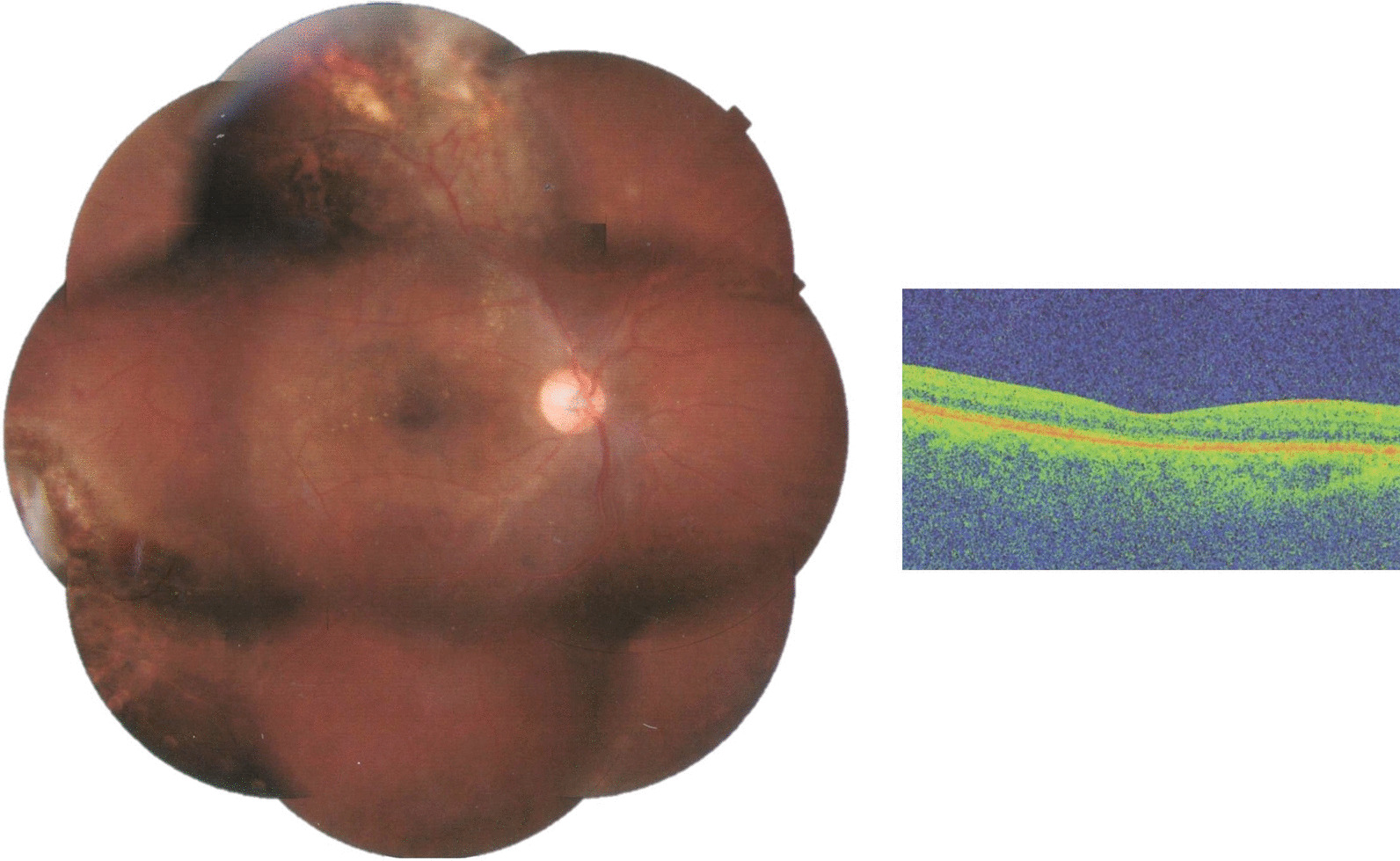
Montage photograph of right eye at the end of last follow-up visit. The retina is completely reattached. The lipid exudation around the macula has subsided and there is a visible scar around the telangiectatic vessel following endophotocoagulation laser. The break in the temporal region is sealed and surrounded by chorioretinal scar. Subsequent SD-OCT of macula reveals resolution of the fluid and exudation with near normal restoration of the foveal anatomy

The amount of subretinal fluid gradually decreased and the patient was discharged on third postoperative day. The patient was advised to remain in a prone position for the next 2 weeks. Two weeks after the surgery, the retina was attached, with BCVA of 1/60. The BCVA gradually improved to 2/60 in the third postoperative week, to 6/60 2 months following the surgery. The postoperative recovery was uneventful and the patient was under regular review. Seven months postsurgery, the BCVA was 5/60 with + 6.00 D spherical lens and there was progression of posterior subcapsular opacification of grade II. He then underwent cataract extraction by phacoemulsification with foldable intraocular lens implantation in the capsular bag, along with silicon oil removal under general anesthesia (Fig. 4). The patient was prescribed with standard postoperative medications as described earlier. Five months after the subsequent surgery (February 2021), the patient was doing well with BCVA of 6/18 and the retina was attached in all quadrants (Fig. 5).

## Discussion

The exact etiology of Coats’ disease is not known. However, mutations in retinal proteins encoded by Crumbs cell polarity complex component (*CRB1*) and Norrie disease pseudoglioma (*NDP*) genes is believed to be the possible pathogenesis [8]. There are two evident pathological processes in the vasculature of Coats’ disease [9]. The first change consists of the breakdown of the blood–retinal barrier at the level of endothelium, which results in plasma leakage and thickening of portions of the vessel wall. These necrotic changes in the vessel wall lead to its disorganization, which is also termed as sausage-like configuration [10]. Second is the degeneration of abnormal pericytes and endothelial cells, causing aneurysms and the closure of vessels leading to ischemia [11]. This process generates lipid-rich exudation into the retina, which can lead to changes in the retina, including thickening, cyst formation, or retinal detachment [5]. These changes are comparable to the changes occurring in diabetic retinopathy [12].

The diagnosis of our case was reached on the basis of clinical examination alone and meticulous exclusion of other probable differentials [2]. The limited resources in our setup precludes us from further genetic testing for confirmation. Ancillary tests such as magnetic resonance imaging and computed axial tomography can be useful diagnostic aids, however, the priority shifted to surgical management in our case. Upon review of the literature, all the features of our case matches those defined for Coats’ disease, which supports our clinical diagnosis. Nonetheless Coats’ disease is believed to have no appreciable retinal or vitreal traction [13]. The rhegmatogenous retinal detachment encountered in Coats’ disease available in the literature has been reported to be of iatrogenic origin following vitrectomy surgery [14], and even after photocoagulation due to traction from preretinal membrane.

There is a paucity of similar reports on rhegmatogenous retinal detachment in Coats’ disease, and we believe this could be the first from this region. A similar report from 1979 was identified [7]. Other than complications of therapeutic modality, retinal break can be the natural course of the disease. There may be a number of factors that might contribute to the formation of retinal break in retinal vascular disorders, including [15] retinal ischemia, retinal exudation, vitreous detachment, vitreous contraction, retinal neovascularization, and underlying choroidal changes. Accelerated senescent vitreous changes at a relatively early age leading to vitreous condensation and vitreous traction might have led to retinal break [7]. Though vitreoretinal traction and fibrosis are rare [13], this could be only attributed to the rhegmatogenous retinal detachment in our case.

The modality of management for Coats’ disease essentially depends upon the stage of the disease. Milder disease with telangiectasia and exudation but not threatening the vision can be observed alone. Laser photocoagulation and cryotherapy are effective options for management [16]. Nonetheless, treatment modalities in Coats’ disease should be tailored individually due to the low incidence of the disease and the great variation in severity upon presentation [9]. Surgery is reserved for advanced cases of detachment. Vitrectomy is advocated for refractory detachment [16]. External drainage of subretinal fluid is generally performed [17], similarly internal drainage is combined with vitrectomy procedure [18]. There does not exist a common consensus for the choice of treatment, surgeon’s discretion based on stage and severity of the disease can influence the management option. We managed the case with encircling band buckle combined with vitrectomy and silicon oil tamponade. The posterior location of the break prompted us for the preference of lens sparing vitrectomy and encircling band to support the vitreous base. Following the subsequent cataractous lens exchange with posterior chamber intraocular lens implantation and silicon oil removal, the useful vision was restored in this patient with good anatomical result. The rapid and complete resolution of subretinal fluid further supported our belief of rhegmatogenous retinal detachment in Coats’ disease [7].

## Conclusion

The presentation of this case with an underlying break not attributed to any iatrogenic modality makes it distinctive. We believe that this case contributes to the existing pool of knowledge in the understanding of Coats’ disease, and timely management of the case restored the best possible anatomical outcome and maintained a good vision. Finally, this study underlines the variable presentation in one of the rarest ocular conditions, and presents a management option that can be adopted in the treatment.

## Data Availability

Data sharing is not applicable to this article as no datasets were generated or analyzed during the current study.

## Abbreviations

RE: Right eye
LE: Left eye
BCVA: Best corrected visual acuity
IOP: Intraocular pressure
SD-OCT: Spectral domain optical coherence tomography
CRB 1: Crumbs cell polarity complex component
NDP: Norrie disease pseudoglioma
